# Diagnosing dementia: No easy job

**DOI:** 10.1186/1471-2296-12-60

**Published:** 2011-06-27

**Authors:** Frank Buntinx, Jan De Lepeleire, Louis Paquay, Steve Iliffe, Birgitte Schoenmakers

**Affiliations:** 1 Katholieke Universiteit Leuven, Department of General Practice, Kapucijnenvoer 33, blok J, B 3000, Leuven, Belgium; 2 Maastricht University, Department of General Practice, PO box 616, Nl 6200 MD Maastricht (the Netherlands; 3 Wit-gele Kruis van Belgie, Ad. Lacomblélaan 69, 1030 Brussels, Belgium; 4 University College London, Department of Primary Care & Population Health, Royal Free Campus, Rowland Hill St., London NW3 2PF UK

## Abstract

**Background:**

From both clinical experience and research we learned that in complex progressive disorders such as dementia, diagnosis includes multiple steps, each with their own clinical and research characteristics.

**Discussion:**

Diagnosing starts with a trigger phase in which the GP gradually realizes that dementia may be emerging. This is followed by a disease-oriented diagnosis and subsequently a care -oriented diagnosis. In parallel the GP should consider the consequences of this process for the caregiver and the interaction between both. As soon as a comprehensive diagnosis and care plan are available, monitoring follows.

**Summary:**

We propose to split the diagnostic process into four diagnostic steps, followed by a monitoring phase. We recommend to include these steps when designing studies on screening, diagnosis and monitoring of patients with dementia and their families.

## Background

Diagnosis is usually thought of as a linear process of performing one or more tests in patients with specific signs or symptoms. If the results show that the patient has the disorder, the diagnostic stage has ended, and treatment can begin. This does not work in primary care, where pattern recognition and the use of 'illness scripts' (more or less complex representations of diseases) are used in non-linear pathways to find the most probable explanations for presenting symptoms [1]. It does not work in specialist care for complex conditions either. If a patient may be developing dementia, the diagnostic process does not start with testing (because of the frequent absence of a clear indication of formal testing) and does not end with applying the treatment logically required by the test results (as also care planning and care for the carers will be required and should regularly be adapted).

In this overview, we combined decades of clinical experience as a GP or a nurse, with the results of a large number of qualitative and quantitative studies. We reached consensus about a number of steps which together build a comprehensive diagnostic work-up in case of cognitive deterioration and dementia. Each of these steps is supported by evidence from primary care-based studies.

### Suspecting the possibility of dementia

Like some other chronic diseases, dementia is a progressive, insidious disorder. There is usually no fixed starting point, the initial clinical picture can be very diverse, and may also mimic other conditions such as normal ageing, depression, or less commonly, a brain tumour. The first stage of diagnosing here is not testing, but starting to suspect the possibility that dementia may be emerging (***the trigger phase***). A specific problem with dementia is that patients, families and general practitioners may all be reluctant to diagnose dementia, a serious and largely untreatable progressive neurodegenerative disease which is very treatening for both patients and families. Physicians may unconsciously hesitate to label a patient as such [2], and family members may gradually take over social roles from the patient, without necessarily being aware of what they are doing. By doing this, they protect the patient from decompensating in daily life, but also delay the conscious recognition of the disorder by offsetting impairments [3].

Triggers for suspecting dementia mostly are subtle signs and symptoms which do not appear in the textbooks and can best be found using qualitative studies. In two such qualitative studies, both carers and physicians were interviewed about the symptom patterns preceding the diagnosis of dementia. The first signs of dementia most frequently noticed by carers or doctors were not the classic 'memory problems'. Suspicion mostly arised from revelatory moments or triggers. Revelatory moments can be any sudden change of environment: hospitalisation, change of medication, or disappearance of the main carer (usually the partner) because of death or hospitalisation. Important triggers for the physician or carers include the development of problems in everyday tasks, behavioural problems, short term memory disorders or failure in work or home tasks [3].

It is not easy for family, carers or nurses who live and work very near to the patient, to correctly describe what they observe and report it to the GP. To help them, observational scales can be used as soon as a problem is identified [4]. These scales only record what has been observed without any interpretation or immediate diagnostic aim. As soon as the GP is informed about the actual concerns, he can decide whether to start a diagnostic process to confirm or refute the presence of cognitive decline.

### A disease-oriented diagnosis

A formal diagnosis of dementia results from a step-by-step and iterative assembly of evidence (a ***disease-oriented diagnosis***). Consecutive test results add to a diagnostic outcome with increasing validity [5]. The suspicion is progressively confirmed (or refuted), one of the dementia subgroups is identified, and other possible reasons for the signs and symptoms are excluded. Diagnosing dementia essentially remains a clinical issue [6] and GPs are mostly well able to correctly diagnose dementia [7]. GPs and specialists perform tests ranging from simple to complex and from highly sensitive to progressively more specific. Cognitive function tests such as the Clock Drawing test, Lawton's IADL test, the 6CIT, the MMSE or one of its validated shorter forms allow a first, sensitive approach that can easily be performed, over a period of time, by the GP [8,9]. Once these initial tests have further increased the likelihood of cognitive impairment, formal testing for a diagnosis of dementia using a standardised test battery such as CAMDEX or ADAS-cog, is indicated. Additional clinical, laboratory and imaging tests can be required to exclude other, treatable disorders, such as depression, visual and hearing problems, endocrine disease or subdural haematoma. Identifying the type of dementia remains a task for a multidisciplinary specialised group [7]. Referral to a memory assessment clinic can provide an opportunity for a comprehensive assessment within a single consultation. The assessment of other co-morbidities which are not directly related to the dementia is essential as either the disorders themselves or treatment may influence the patient's functional status.

### Assessing care needs

Usually, a formal diagnosis of one type of dementia does not provide us with all the information we need. There is no clear-cut disease-modifying treatment for dementia [10], and people with dementia tend to survive for an average of five years after diagnosis [11,12], making care more important than cure. Within this framework, it is good to mention that - contradictory to what is sometimes thought - disease-specific methods have been developed to measure and monitor quality of life, also in demented people. Progression of cognitive decline is thereby not necessarily related to decreasing quality of life [13,14]. Therefore, careful and repeated assessment of the patient's care needs is essential to guide the efforts of all those helping the patient over a period of many years (***care-oriented diagnosis***). A number of assessment methods have been developed, of which the Resident Assessment Instrument (MDS-RAI) and the Camberwell Assessment of Need for the Elderly (CANE) seem the most helpful [15,16].

Listing care needs does not help much, however. Care needs have to be translated into care goals, and then the steps to be taken or tasks to be performed to reach these goals have to be planned [17]. Such planning is best performed during meetings where all members of the care team take responsibility for part of the tasks [18]. In our experience, such team meetings are far more effective if the results of a formal assessment of the patient's care needs, as provided by the MDS-RAI, are available.

### Including carers in your diagnostic approach

In most cases people with dementia do not live alone. Family members may play an important role as carers. However, these carers will develop their own needs, which frequently go undetected and neglected. The risk of depression in non-professional carers of demented people is far higher than in other people of the same age and sex [19]. In particular, behavioural disturbances are responsible for a large part of the distress [20]. The subjective burden which is highly related to the coping behaviour of the carer [19,21], is more predictive for the risk of depression than the objective burden of tasks to be performed. Assessing the carer's needs (***carer assessment***) requires continuous attention, especially from the GP, who is best placed to monitor the sometimes unsteady balance between the burden and the coping strategies of the carer. A large number of interventions to support carers have been tested using simple (patient level) or clustered (GP or practice level) randomized clinical trials. Simple and straightforward interventions [22-25] are the most appealing and many of them have been tested. In general, satisfaction of patients and carers with such interventions is high. However, if more robust outcome measures such as depression in carers or time to institutionalisation are used, almost no effect is found [24]. Recently, in a small scale RCT, a more comprehensive intervention was tested including a care manager who made some formal visits to the carer and was continuously within reach by phone. In the intervention group, incidence of depression in carers was significantly reduced to 16% compared to the control group [26].

### Monitoring

Dementia is a chronic disorder with consequences for both patient and carers changing over time. **Monitoring **these changes and adapting care accordingly is therefore necessary. Literature on the optimal frequency and methods for such monitoring is scarce, not to say almost inexistent. In the recently published and outstanding book on evidence-based monitoring [27] dementia is not even an index term. There is much scope for research in this field.

## Conclusions

**Figure 1:**
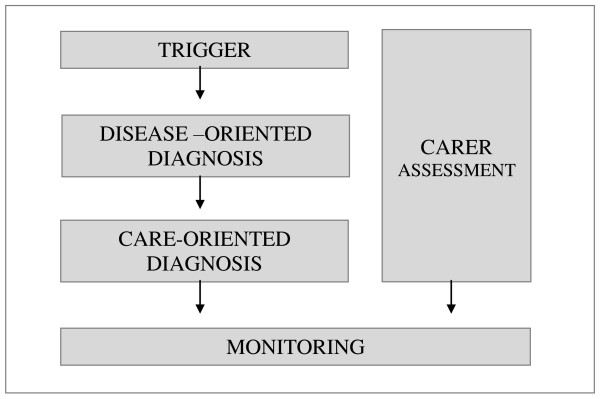
**Stages in diagnosing dementia**.

Diagnosing dementia is far more complicated than just performing a few tests and deciding whether a patient has dementia or not. We therefore proposed splitting the diagnostic process into four diagnostic steps, followed by a monitoring phase: the trigger phase, a disease-oriented diagnosis, a care-oriented diagnosis and carer assessment (figure 1). The GP, together with other professional and non-professional carers, has many questions to answer and assessments to perform before finally implementing a strategy that provides optimal and comprehensive help in order to retain capacities and maximise quality of life of all people included. It may be important to take all these steps into consideration when designing studies to identify strengths and weaknesses of interventions intended to improve screening, diagnosis and monitoring of patients with dementia and their families.

## Competing interests

The authors declare that they have no competing interests.

## Authors' contributions

FB took the initiative for this paper. He co-ordinated the discussions and drafted the manuscript. FB, JDL, LP, SI and BS performed part of the research which lead to this paper. They took part in the discussions, read and commented upon the different drafts and approved the final version.

## Pre-publication history

The pre-publication history for this paper can be accessed here:

http://www.biomedcentral.com/1471-2296/12/60/prepub
